# Global gene network exploration based on explainable artificial intelligence approach

**DOI:** 10.1371/journal.pone.0241508

**Published:** 2020-11-06

**Authors:** Heewon Park, Koji Maruhashi, Rui Yamaguchi, Seiya Imoto, Satoru Miyano

**Affiliations:** 1 M&D Data Science Center, Tokyo Medical and Dental University, Tokyo, Japan; 2 Fujitsu Laboratories Ltd, Kanagawa, Japan; 3 Division of Cancer Systems Biology, Aichi Cancer Center Research Institute, Aichi, Japan; 4 Human Genome Center, The Institute of Medical Science, The University of Tokyo, Tokyo, Japan; University of Vermont, UNITED STATES

## Abstract

In recent years, personalized gene regulatory networks have received significant attention, and interpretation of the multilayer networks has been a critical issue for a comprehensive understanding of gene regulatory systems. Although several statistical and machine learning approaches have been developed and applied to reveal sample-specific regulatory pathways, integrative understanding of the massive multilayer networks remains a challenge. To resolve this problem, we propose a novel artificial intelligence (AI) strategy for comprehensive gene regulatory network analysis. In our strategy, personalized gene networks corresponding specific clinical characteristic are constructed and the constructed network is considered as a second-order tensor. Then, an explainable AI method based on deep learning is applied to decompose the multilayer networks, thus we can reveal all-encompassing gene regulatory systems characterized by clinical features of patients. To evaluate the proposed methodology, we apply our method to the multilayer gene networks under varying conditions of an epithelial–mesenchymal transition (EMT) process. From the comprehensive analysis of multilayer networks, we identified novel markers, and the biological mechanisms of the identified genes and their reciprocal mechanisms are verified through the literature. Although any biological knowledge about the identified genes was not incorporated in our analysis, our data-driven approach based on AI approach provides biologically reliable results. Furthermore, the results provide crucial evidences to reveal biological mechanism related to various diseases, e.g., keratinocyte proliferation. The use of explainable AI method based on the tensor decomposition enables us to reveal global and novel mechanisms of gene regulatory system from the massive multiple networks, which cannot be demonstrated by existing methods. We expect that the proposed method provides a new insight into network biology and it will be a useful tool to integrative gene network analysis related complex architectures of diseases.

## Introduction

Gene regulatory networks are crucial for understanding complex mechanisms of diseases. To reveal heterogeneous genetic networks that underlie complex human diseases, various large-scale projects (e.g., The Cancer Genome Atlas and Cancer Genome Project) have been conducted and provided considerable amounts of omics data. The scale of gene networks is increasing, and strategies to comprehensively analyze large-scale gene networks have been claimed. In particular, there is currently substantial discussion regarding integrative analysis of sample-specific gene networks for personalized cancer diagnostics and therapeutics.

Shimamura et al. [1] proposed a statistical method for sample-specific network construction, NetworkProfiler, which groups samples according to their specific genomic characteristics (e.g., drug response and survival time) and constructs a network for a target sample based only on samples having characteristics similar to those of the target sample. Thus, we can reveal gene regulatory networks under varying conditions of clinical characteristics of patients. The NetworkProfiler was applied to construct gene networks for 762 cancer cell lines characterized by EMT process, where EMT-related modulators for each cell line were measured based on 50 EMT-related genes labeled in the Molecular Signatures Database. They focused on E-cadherin, which connects epithelial cells at adherens junctions, and identified 24 candidate regulators. Interestingly, the identified genes did not consist of just the 50 genes defining the modulators, i.e., only one of the 24 identified genes was a member of the 50 genes, even though the regulators of E-cadherin were identified from the networks under varying conditions of the EMT modulators computed from the 50 genes. Among the 24 identified genes, approximately half were verified as regulators of E-cadherin from the literature. They selected KLF5 from the remaining genes and performed validation experiments. Through the experiments, the mechanism of KLF5 was demonstrated: knockdown of KLF5 decreased the expression of E-cadherin and led to morphological changes of the characteristics of EMT. Their validation results are also supported by later studies, e.g., Zhang et al. [2]. Although the other half were not verified at that time, a majority of those have been demonstrated as crucial EMT markers in the past decade [3, 4]. Shimamura et al. [1] provided crucial indicators and made major contributions to reveal tumor progress related to EMT. Park et al. [5] suggested that cancer characteristics are not uniformly distributed, and the Gaussian kernel function used to control the effect of samples in the NetworkProfiler leads to extremely small amount of weight for modeling a target sample having rare cancer characteristic, because the Gaussian kernel function is based on a constant bandwidth. To address this problem, Park et al. [5] proposed a robust version of NetworkProfier based on an adaptive bandwidth via the k-nearest neighbor rule, and constructed a drug sensitivity-specific gene network based on the Sanger dataset from the Cancer Genome Project.

Although existing studies have provided crucial tools for precision medicine, understanding of large-scale multilayer gene networks is limited. In other words, a significant number of multilayer networks cannot be interpreted comprehensively using existing approaches (e.g., Shimamura et al. focused only on E-cadherin, and other regions could not be revealed).

To resolve this problem, we propose a novel strategy based on an explainable artificial intelligence (AI) methodology using tensor decomposition. Although machine learning and AI methods show remarkable performance in modeling accuracy, most of the existing approaches cannot explain how they obtain results (referred to as the black-box problem). This limits AI usage because their results cannot be verified. Maruhashi et al. [6, 7] developed novel explainable AI approaches (i.e., DeepTensor and Tensor Reconstruction-based Interpretable Prediction (TRIP)) for learning multiway relations, which are deep learning approaches using tensor decomposition. Our strategy is based on two stages, i.e., constructing sample-specific gene networks and comprehensive analysis of the constructed multilayer netowrks by using the explainable AI methodology. That is, we construct a personalized gene regulatory network for each patient and the constructed network is considered as a second-order tensor. We then explore the massive multiple gene networks by using the AI method, TRIP. The use of the interpretable AI method based on tensor decomposition enables us to overcome limitation of existing gene network analysis, i.e., narrow angle in regulatory networks, and this leads to a greater understanding of biological systems of the regulatory interactions between genes. To the best of our knowledge, this is the first study on revealing gene regulatory networks using an explainable AI method.

To illustrate our strategy, we apply the proposed method to EMT-related gene regulatory networks constructed using NetworkProfiler [1]. We learn a low-dimensional subspace of 762 network tensors using the TRIP and explore multilayer networks on the constructed subspace. From the comprehensive analysis of multilayer networks, we identified novel markers, and the biological mechanisms of the identified genes and their reciprocal mechanisms are verified through the literature. Although any biological knowledge about mechanism of the identified genes was not incorporated in our analysis, the revealed genes by our method have strong evidences. In other words, our data-driven approach provides biologically reliable results. Although we illustrate our method based only on EMT-related networks, it can be expected that the proposed method will be a useful tool to global explore gene regulatory system involved in various clinical characteristics.

The remainder of this paper is organized as follows. In the Method section, we introduce the proposed a novel strategy for global exploration of multilayer personalized gene networks. In the Results section, we describe the evaluation of our strategy based on the gene regulatory networks varying according to the EMT status. Conclusions are provided in the Discussion section.

## Method

Suppose *X*_1_, …, *X*_*q*_ is *q* possible regulators that may control transcription of the *l*^*th*^ target gene *Y*_*l*_. Consider the linear regression model for the target gene *Y*_*l*_,

$$\begin{array}{c} Y_{l}=\sum_{j=0}^{q}\beta_{jl}\cdot X_{j}+\varepsilon_{l}, \end{array}$$ (1)

where *β*_*jl*_ is the regression coefficient that represents the effect of regulator *X*_*j*_ on target *Y*_*l*_ and *ε*_*l*_ is a random error vector *ε*_*l*_ = (*ε*_*l*1_, …, *ε*_*ln*_)^*T*^ that is assumed to be independently and identically distributed with mean 0 and variance *σ*^2^. To reveal gene regulatory interactions based on the regression model, various statistical and machine learning methods have been proposed and applied to gene network construction [8, 9]. However, patient-specific gene regulatory systems cannot be revealed via the regression model because the strengths of the relationships between genes are given as *β*_*jl*_ for all samples.

We develop a novel strategy for integrative analysis for multilayer gene networks, which are a crucial tool for precision medicine. In our method, gene regulatory networks are constructed under varying conditions of samples and the multilayer networks are analyzed comprehensively using an explainable AI method. The gene network for a target sample is considered as a second-order tensor, and a deep learning method for tensor decomposition is applied to construct low-dimensional subspace of the multiway interaction between genes. Prediction and interpretation are performed on the constructed human-readable low-dimensional subspace, and thus we can effectively understand the constructed large-scale gene networks. Our strategy consists of two stages of constructing sample-specific gene regulatory networks and global investigating large-scale multiple gene networks based on an explainable AI technology.

### Stage 1: Constructing personalized gene regulatory network based on sample-specific analysis

We consider the varying-coefficient structural equation model to construct a sample-specific gene regulatory network [10],

$$\begin{array}{c} Y_{l}=\sum_{j=0}^{q}\beta_{jl}\left(m_{\alpha }\right)\cdot X_{j}+\varepsilon_{l}, \end{array}$$ (2)

*β*_*jl*_(*m*_*α*_) is the regression coefficient of *X*_*j*_ on *Y*_*l*_ for the *α*^*th*^ target sample of the modulator *M* = *m*_*α*_, such as drug sensitivity and survival risk of cell lines.

To estimate the varying coefficient *β*_*jl*_(*m*_*α*_) describing strength of the relationship between the regulator and the target genes for each sample, we considered the the following kernel-based L1-type regularization method [1, 5]

$$\begin{array}{c} L\left(\beta_{l\alpha }|b_{l}\right)=\frac{1}{2}\;\sum_{i=1}^{n}{\left\{y_{il}\;-\;\sum_{j=1}^{q}\beta_{jl\alpha }X_{ij}\;\right\}}^{2}G\left(m_{i}\;-\;m_{\alpha }|b_{l}\right)\;+P\left(\beta_{l\alpha }\right), \end{array}$$ (3)

where *P*(***β***_*lα*_) is the recursive elastic net penalty, and

$$\begin{array}{c} G\left(m_{i}-m_{\alpha }|b_{l}\right)=\text{exp}\{\frac{-{\left(m_{i}\;-\;m_{\alpha }\right)}^{2}}{b_{l}}\} \end{array}$$ (4)

is the Gaussian kernel function used to group samples according to specific cancer characteristics (i.e., modulator *m*_*i*_ for *i* = 1, …, *n*). The Gaussian kernel function enables us to estimate *β*_*jl*_(*m*_*α*_) for the *α*^*th*^ sample based only on samples having characteristics of *m*_*i*_ similar to the target sample modulator value *m*_*α*_. Thus, we can construct a gene network for a specific clinical status of samples, and it leads to evidences for personalized therapy.

In the first stage, we measure cancer characteristics *m*_*i*_ (*i* = 1, …, *n*) of each sample and construct personalized gene regulatory networks under varying conditions of clinical characteristics using NetworkProfiler. This enables us to reveal patient-specific gene regulatory characteristics that are vital information of precision medicine. To comprehensively analyze the multiway interaction between genes, we proposed the use of the AI method in the second stage.

### Stage 2: Extracting knowledge from the multilayer networks by explainable AI

The constructed multilayer networks (targets × regulators × samples) are considered as the input of the explainable AI method developed in our previous study, called TRIP [7]. The TRIP is a deep learning method for tensor decomposition. In this study, we consider a gene network matrix as a second-order tensor for a data point and then estimate projection matrices for the first and second modes based on the tensor decomposition. By using the projection matrices, we construct low-dimensional subspace of the network tensor. Thus, we can reduce dimensionality of large-scale multiple gene networks and extract crucial components to predict EMT-modulators.

For the *K*-mode tensor X for size *I*_1_ × ⋯ × *I*_*K*_, the TRIP estimates a projection matrix $C^{\left(k\right)}\in R^{I_{k}\times J_{k}}$ and then projects the network tensors onto the constructed subspace by using ***C***^(*k*)^. Prediction or classification is conducted on the constructed human-readable low-dimensional subspace. This leads to more explainable and interpretable results of the multilayer gene network analysis, as compared to the results on the complex high-dimensional data space.

The second stage of our strategy is based on the following two problems,

- Constructing human-readable low-dimensional subspace and projecting the network tensor onto the subspace,
  $$\begin{array}{c} {\bar{X}}_{i}=X_{i}\prod_{k}\times_{k}C^{\left(k\right)} \end{array}$$ (5)
- Predicting a response variable based on the projected network tensor ${\bar{X}}_{i}$
  $$\begin{array}{c} {\hat{y}}_{i}=f\left({\bar{X}}_{i},\theta \right)\;\text{for}\;i=1,\ldots ,n, \end{array}$$ (6)
  where $r_{i}=W_{i}^{T}\text{vec}\left({\bar{X}}_{i}\right)$, $W_{i}$ is the weight tensor for prediction and ***θ*** are the remaining parameters other than $W_{i}$.

The optimization of the TRIP for the two aforementioned problems (i.e., projection of the network tensor and prediction of the response variable) is based on the following objective function

$$\begin{array}{c} O_{T}=\frac{1}{n}\sum_{i=1}^{n}\{L\left(y_{i},{\hat{y}}_{i}\right)+\gamma \|X_{i}-{\bar{X}}_{i}\prod_{k}\times_{k}{\text{C}}^{(k)T}{\|}_{2}^{2}\},\\ \text{subject}\;\text{to}\;{\text{C}}^{(k)T}{\text{C}}^{\left(k\right)}=\text{I}, \end{array}$$ (7)

where *γ* > 0 is the tuning parameter for the projection error and $L(y_{i},{\hat{y}}_{i})$ is the loss function of the prediction given in (6). The second term is the loss function for projection of the network tensor onto the constructed subspace given in (5). As shown in the objective function (7), the TRIP estimates the projection matrix ***C***^(*k*)^ and simultaneously predicts the response variable. This implies that the TRIP enables us to achieve effective prediction results while retaining as much of the original data variance as possible.

The projection matrix ***C***^(*k*)^ that satisfies the orthonormal condition (i.e., **C**^(*k*)*T*^ **C**^(*k*)^ = **I**) is derived from singular value decomposition (SVD) of a latent variable ***Z***^(*k*)^ of the same size as ***C***^(*k*)^. That is, we first perform SVD of ***Z***^(*k*)^,

$$\begin{array}{c} Z^{\left(k\right)}=P^{\left(k\right)}S^{\left(k\right)}Q^{(k)T}, \end{array}$$ (8)

and then set

$$\begin{array}{c} C^{\left(k\right)}=P^{\left(k\right)}Q^{(k)T}. \end{array}$$ (9)

The latent variable ***Z***^(*k*)^ is estimated from the derivatives of the objective function *O*_*T*_ by setting them to zero. Maruhashi et al. [7] showed that the derivatives of the objective function *O*_*T*_ with respect to *Z*^(*k*)^ are derived from a function of ∂*O*_*T*_/∂*C*^(*k*)^ and SVD of *Z*^(*k*)^, and proposed iterative algorithm for optimization problem of the TRIP given in (7). That is, the optimization problem of the TRIP is based on simultaneously taking the derivatives of the objective function *O*_*T*_ with respect to ***C***^(*k*)^, ***Z***^(*k*)^, ***r***_*n*_, and ***θ*** and setting them to zero.

The projection matrices are considered as the regression coefficients of the crucial components. They learnt a multi-linear surrogate model ${\hat{y}}_{i}^{\prime }=\langle W,{\bar{X}}_{i}\rangle +b$ that minimizes the sum of the differences with the results of the prediction model $\sum_{i}{\left\|{\hat{y}}_{i}-{\hat{y}}_{i}^{\prime }\right\|}_{2}^{2}$, by setting the regression coefficient W to be a multi-linear tensor, i.e., $W=g^{\left(1\right)}\circ \cdots \circ g^{\left(K\right)}$, where ∘ denotes outer product. The crucial components in prediction of response variable are extracted by principal component analysis of the vectors

$$\begin{array}{c} u_{i}^{\left(k\right)}=X_{i}\prod_{l|l\neq k}C^{\left(l\right)}g^{\left(l\right)}\times C^{\left(k\right)} \end{array}$$ (10)

That is, the principal components of ***U***^(*k*)^ having the normalized $u_{i}^{\left(k\right)}$ as the column vector are extracted as crucial components for prediction of the response. For details of the notation and algorithm for the TRIP, please refer to Maruhashi et al. [7].

In the second stage, we perform integrative gene network analysis based on the constructed subspace of the network tensors.

## Results

To illustrate our strategy, we apply the proposed method to personalized gene regulatory networks varying depending on the EMT process [1]. Because the EMT modulator is uniformly distributed, we consider EMT-related networks constructed by ordinary Networkprofiler instead of the robust NetworkProfiler.

### Personalized gene regulatory networks under varying conditions of the EMT process

EMT-related gene networks were constructed based on the expression profiles of 762 cell liens from the Sanger Cell Line Project (http://www.broadinstitute.org/cgi-bin/cancer/datasets.cgi). A 13,508 (genes) × 762 (cancer cell lines) gene expression matrix was constructed based on the expression profiles of 13,006 mRNAs from Affymetrix GeneChip Human Genome U133 Array set and 502 microRNAs from bead-based oligonucleotide arrays. A total of 1732 regulator genes consisting of 1183 transcription factors, 47 nuclear receptors, and 502 human miRNA were extracted. The modulator describing the EMT process (epithelial-like or mesenchymal-like) of cell lines, the so-called EMT modulator, was extracted using the module discovery method [11] based on 50 genes labeled as the EMT-related genes (i.e., EMT-UP, EMT-DN, JECHLIN-GER-EMT-UP, and JECHLIN-GER-EMT-DN) in Molecular Signatures Database v2.5 (http://www.broadinstitute.org/gsea/msigdb/index.jsp). In short, 762 EMT-related gene regulatory networks for 13,508 targets with 1732 regulator genes were constructed under varying conditions of the EMT modulator values. In this study, we consider the network for 13,508 targets × 1762 regulators as a second-order tensor for a specific EMT process and apply the TRIP for integrative gene network analysis of the 762 network tensors.

### Comprehensive interpretation of the EMT-related network tensors

**Fig 1:**
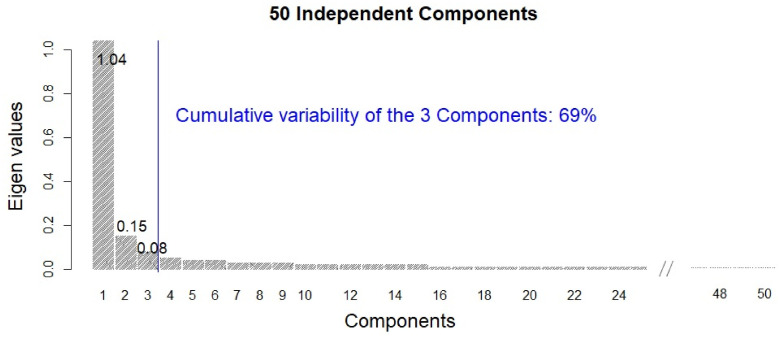
Variabilities of the 50 independent components.

We learn a 50 × 50 subspace of the 762 EMT-related gene networks using the TRIP, i.e., *J*_1_ = 50 and *J*_2_ = 50. From the projection matrices of the constructed subspace ***C***^(*k*)^ *k* = 1, 2 in (9), each 50 crucial factors describing importance of target and regulator genes to predict EMT-modulator are extracted by PCA of ***U***^(1)^ and ***U***^(2)^, respectively. In this study, we consider the crucial components of the subspace for regulator genes, i.e., ***U***^(2)^. Fig 1 shows the variabilities explained by the extracted 50 independent components. As shown in Fig 1, the first component explains more than half of the total variability (i.e., 56%) of the EMT-related gene networks, and the first three components explain approximately 70% of the variability. We focus on the first three components and interpret the EMT-related gene networks based on the three components.

Table 1 shows the distribution of the tissue origin of 100 cell lines corresponding to the 100 highest and lowest values of each of the three components. The brain cell lines are concentrated in the region with high values of the first component (i.e, 83% of brain cell lines are in the region with the 100 highest values of component 1), and leukemia cell lines are concentrated in the region with high values of the second component (i.e, 83% of leukemia cell lines are in the region with the highest values of component 2).

**Table 1:** Distribution of diseases for cells corresponding the 100 highest and lowest three components (%).

	Component 1			Component 2			Component 3			Total 762 cells (#)
	High	Low	*χ*^2^	High	Low	*χ*^2^	High	Low	*χ*^2^	
AdrenalGland	0	0		100	0		0	0		1
AutoGanglia	5	0	****	3	0		0	54	***	37
BiliaryTrack	17	17		0	33		0	0		6
Bone	29	0	****	3	6		10	13		31
Brain	83	0	****	0	17	****	41	5	****	59
Breast	7	26	***	10	17	**	12	10		42
Cervix	0	43		21	36		7	14		14
Colorectal	0	63	****	5	63	***	8	5	****	38
Endometrium	9	9		0	9		0	18		11
Eye	0	0		100	0		0	0		2
Headneck	4	13	***	0	8		33	0		24
Kidney	9	0	****	0	0		5	32		22
Leukemia	0	1	****	50	1	****	16	0	****	109
Liver	18	0		18	0		9	9		11
Lung	2	15	**	8	12		9	15	**	128
Lymphoma	0	0		32	0	****	16	0		19
Muscle	0	0		0	13		0	63		8
Oesophagus	0	38	***	0	33	***	4	8		24
OtherSarcoma	70	0	**	0		0	20	10		10
Ovary	4	0		33	0		17	21		24
Pancreas	0	41		6	29		12	0		17
Placenta	0	0		0	0		0	0		2
Pleura	33	0		0	0		0	17		6
Prostate	0	17		0	17		17	0		6
Skin	26	2	***	0	7		7	33		46
SmoothMuscle	0	0		0	0		0	100		1
StomachGI	0	28		12	24		12	8		25
Testis	0	0		0	0		50	0		4
Thyroid	42	8		0	8		8	0		12
Unknown	0	0		100	0		0	0		1
UrinaryTrack	5	40		0	30		10	15		20
Vulva	0	0		0	0		50	50		2

Significant of *χ*^2^ test (p-value):

** *p* < .01;

*** *p* < .001;

**** *p* < .0001

**Fig 2:**
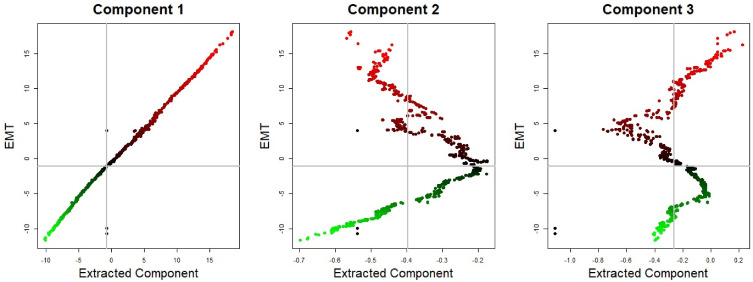
EMT modulator and top three components (Green: low EMT modulator, red: high EMT modulator). The first three components seem to be independent of each other, i.e., each component may have different information to describe EMT-related gene networks.

Colorectal cell lines are concentrated in the region with low values of the first two components. In short, the first and second components can be characterized by the ‘Brain with Colorectal’ and ‘Leukemia with Colorectal’, respectively. An independent test (i.e., Chi-squared test) between high/low values of each component (e.g., leukemia cells/nonleukemia cells and high/low values of each component) is conducted, and significant diseases (*p* < .01) for each component are given in the column *χ*^2^ in Table 1, where high and low regions are classified based on the median value of each component. The brain seemed to be most associated with the first component, whereas Leukemia can be considered as the most significant disease for both the second and third components. The Brain, Leukemia and Colorectal seem to be crucial diseases for all three components. Fig 2 shows the scatter plot of the EMT modulator and extracted three components. As shown in Fig 2, the first component is strongly associated with the EMT modulator (i.e., the correlation coefficient of the EMT modulator and the first component is 0.997). This indicates that the extracted first component can be considered as another EMT modulator, and the second and third components may have information other than the EMT-related mechanism.

In order to globally explore the EMT-related mechanisms from the multilayer networks, we combine our results with the well-known EMT markers (i.e., five EMT transcription factors (EMT-TFs): ZEB1, ZEB2, SNAIL1, SNAIL2, and TWIST1, see Table 2). For each component, the target networks of the five EMT-TFs are constructed in the region of high and low values of each component. From the multiple networks in each region, we first extract target genes (TG.EMT-TFs) of the EMT-TFs and then extracted the target genes of the TG.EMT-TFs, where the genes are extracted as targets in at least one network are considered as target genes. The target networks for each region are give as binary adjacency matrices (1: two genes are associated in at least one cell line, 0: otherwise). Next, we compute compute absolute differences of the two adjacency matrices for genes, then extract 10 genes showing considerably different edges between the two adjacency matrices for high and low regions of each component (i.e., for each genes, sum of the absolute differences are computed for all genes in the EMT target network, and then 10 genes having the largest sum of the absolute differences are extracted). The identified 10 genes can be considered as markers having specific regulatory characteristics depending on the each component.

**Table 2:** EMT Transcription Factors (TFs) and their mechanisms.

EMT-TFs	EMT-related mechanism	Evidences
ZEB family: ZEB1, ZEB2	: Snail1 and Twist1 can up-regulate and cooperate with ZEB1 to induce EMT. : MYC-or serum-induced EMT were characterized by increased expression of ZEB1, ZEB2, and SNAI1.	[3, 12]
SNAIL1	: mediator in different signaling pathways that induce EMT, such as the NBS1-SNAIL1 axis and the TGF-*β*/SMADS/HMGA2/SNAIL1 axis. : main role in EMT, the process by which epithelial cells acquire a migratory, mesenchymal phenotype as a result of its repression of E-cadherin.	[13, 14]
SNAIL2	: up-regulated by Notch to induce EMT with an increase of cell migration and loss of cell–cell junctions. : activates ZEB1 and cooperates with it to promote EMT. : direct induction of SNAIL1 is essential for TWIST1 to induce EMT.	[4, 15, 16]
TWIST1	: HIF-1*α* directly binds to the promoter of TWIST1 to induce EMT in hypoxic microenvironments. : needs to induce SNAIL1 to suppress the epithelial branch of the EMT program. : acts together with SNAIL1 to promote EMT.	[4, 17]

Table 3 shows the identified genes for the three components and their evidence sources. We focus only on the newly identified genes other than the five EMT-TFs, even though the five EMT-TFs are selected for all three components.

**Table 3:** Identified novel candidate markers involved in EMT related mechanism.

Genes	Components	Related diseases	Reference
AFF1	3	-	
ANKRD5	1	Brain, Pharynx, and Swim bladder cancers	[18]
FOXF1	2	Colorectal and Lung cancers	[19, 20]
FOXF2	1	Basal-like breast cancer	[21, 22]
GLI3	2	Oral squamous cell carcinoma and Colorectal cancer	[23, 24]
GRHL2	1	Gastric, Ovarian, and Breast cancers	[13, 25–28]
IFI16	2, 3	Prostate Cancer	[29–32]
IRF6	3	Embryonic palate, Breast, Gastric, and Prostate cancers	[33, 34]
KANK2	3	-	
LSR	2	Endometrial and Breast cancers as well as Head and Neck Squamous Cell Carcinomas	[35–38]
MAFB	3	-	
OVOL2	2	Colorectal tumor, Osteosarcoma, and Breast and Prostate cancers	[39–42]
PCBD1	2	-	
SOX13	2	Colorectal cancer	[43]
TGFB1I1	2	Lung adenocarcinoma, Kidney disease, and Renal fibrosis	[44–47]
TP63	2, 3	Breast cancer	[48–50]
ZNF91	1	-	

Among the newly identified 17 genes, only IRF6 is used for defining the EMT modulator. For the identified genes, we compute the regulatory effect change (REC) for 13,508 target genes [1, 5]. In this study, we consider direct connection between genes, i.e., in the 762 EMT-related gene regulatory networks, the effect of the *j*^*th*^ regulator on the *l*^*th*^ target gene at the *α*^*th*^ sample can be measured by the following regulatory effect (RE),

$$\begin{array}{c} {\text{RE}}_{jl\alpha }={\hat{\beta }}_{jl}\left(m_{\alpha }\right)\cdot X_{\alpha j}, \end{array}$$ (11)

and the RECs according to the EMT modulator values are computed as follows

$$\begin{array}{c} {\text{REC}}_{jl}=\text{max}\left\{{\text{RE}}_{jl\alpha };\alpha =1,...,n\right\}-\text{min}\left\{{\text{RE}}_{jl\alpha };\alpha =1,...,n\right\}. \end{array}$$ (12)

which measures the effect of the EMT modulator on strength of the relationship between the regulator and the target genes.

**Fig 3:**
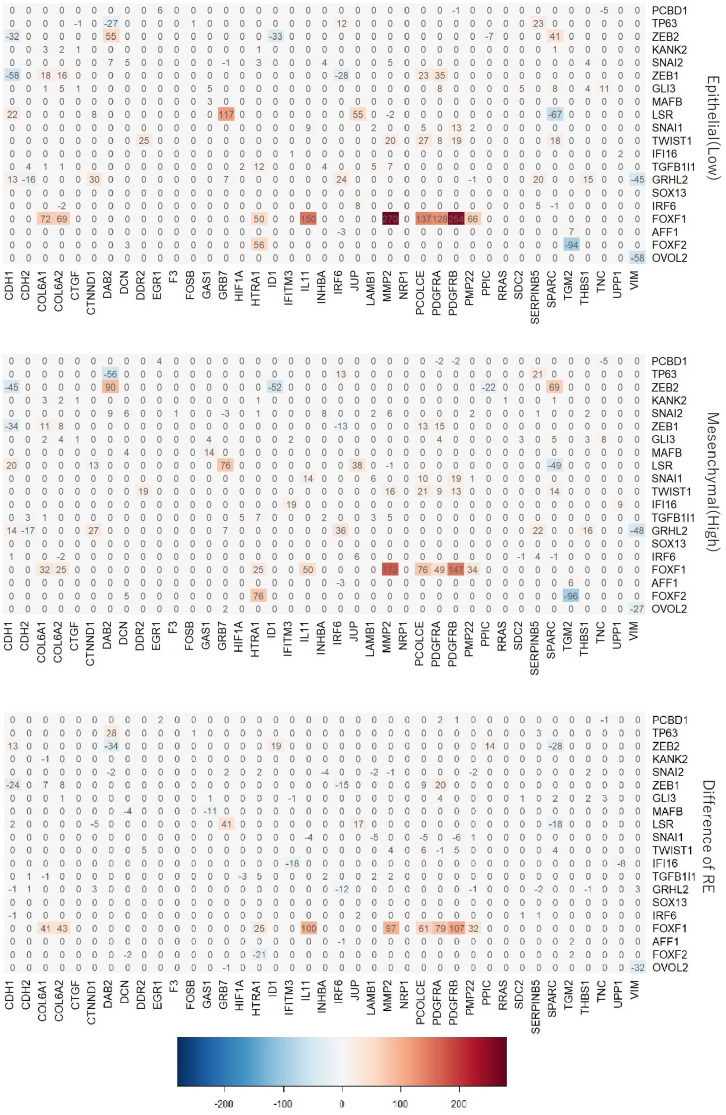
HeatMap of the regulatory effect of the selected genes on the 50 EMT-related genes.

As mentioned above, each 762 cell lines have EMT modulator values and corresponding networks. Fig 3 shows the average of RE of the identified genes other on the 50 EMT-related genes for 10 networks corresponding to the 10 highest (top) and lowest (middle) EMT modulator values, where edges having zero mean for 10 cell lines are deleted. As shown in Fig 3, FOXF1 regulates the EMT-related genes, COL6A1, COL6A2, HTRA1, IL11, MMP2, PCOLCE, PDGFRA, PDGFRB, and PMP22. The regulation system of FOXF1 with the EMT-related genes is observed in both epithelial-like and mesenchymal-like cell lines. E-cadherin (CDH1), which is one of the important genes for cell–cell adhesion in epithelial cells, is positively regulated by LSR and GRLH2 and negatively regulated by the ZEB family (i.e., ZEB1 and ZEB2). We then focus on genes not discovered as EMT markers in the literature, but selected by our method, AFF1, KANK2, PCBD1, ZNF91, and MAFB, i.e., their EMT-related mechanisms have not yet been revealed. Although a majority of the unrevealed genes regulate the EMT-related genes, their REs are extremely small. From these results, it can be considered that the genes may have mechanisms that are not directly involved in the EMT process.

**Fig 4:**
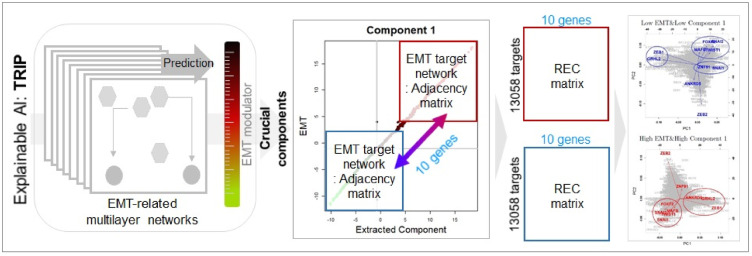
Overall flowchart of our strategy.

For the selected 10 genes from the analysis of the three components, we compute REC matrices consisting of 10 columns (extracted 10 genes) and 13,493 rows (13,493 target genes) and then perform PCA of the REC matrix. The identified genes for each component are grouped in the first two PC spaces, and the reciprocal mechanisms between the grouped genes can be found in the published literature. Overall flowchart of our strategy for global exploration of EMT-related networks is given in Fig 4.

**Component 1**:

**High.E&High.C1 versus Low.E&Low.C1**

**Fig 5:**
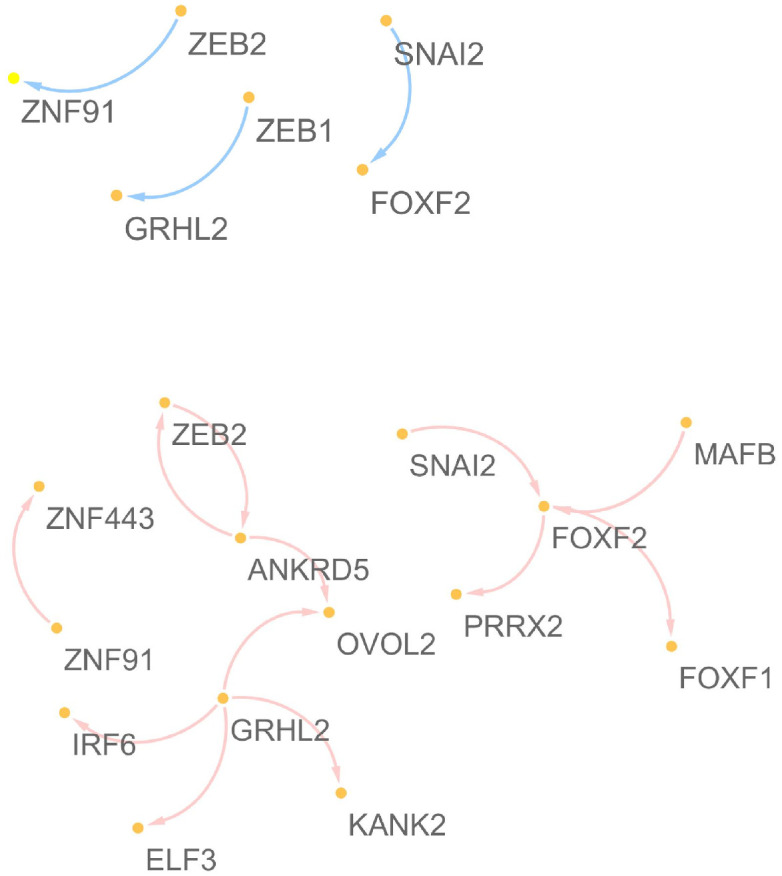
Target networks of the five regulators discovered from the analysis of the first component.

We consider high EMT&high component 1 (High.E&High.C1) and Low EMT&Low component 1 (Low.E&Low.C1) regions consisting of 380 and 379 cell lines, respectively. For the first component, ZEB2, SNAI2, ZEB1, SNAI1, TWIST1, MAFB, GRHL2, ANKRD5, FOXF2, and ZNF91 are selected as markers having specific characteristics depending on the first component (i.e., these genes show significantly different regulatory systems between the High.E&High.C1 and Low.E&Low.C1 regions). In addition to the EMT-TFs, the novel five genes are identified, i.e., MAFB, GRHL2, ANKRD5, FOXF2, and ZNF91. Fig 5 shows the target networks of the five genes. A relatively sparse network can be seen in the Low.E&Low.C1 region, as compared to the High.E&High.C1 region. That is, only five EMT-TFs have a strong relationship between each other in Low.E&Low.C1, whereas there are many relationships between not only the identified 10 genes, but also their target and regulator genes in the High.E&High.C1 region. As shown in Table 3, more than half of the identified genes are confirmed as EMT markers (i.e., their EMT-related mechanisms have been reported in the literature). For instance, GRHL2 reduces the invasion and migration through the inhibition of TGF-*β*-induced EMT in gastric cancer [26]; GRHL2 leads to mesenchymal–epithelial transition (MET) through the inhibition of ZEB1 [13]. ANKRD5 plays roles in protocadherin-mediated cell protrusion and adhesion, and participates in cell adhesion [18]. FOXF2 was identified as a novel TF related to EMT-suppressing in basal-like breast cancer (BLBC) FOXF2 negatively targets TWIST1 in the EMT programming and metastasis progress of BLBC [22].

**Fig 6:**
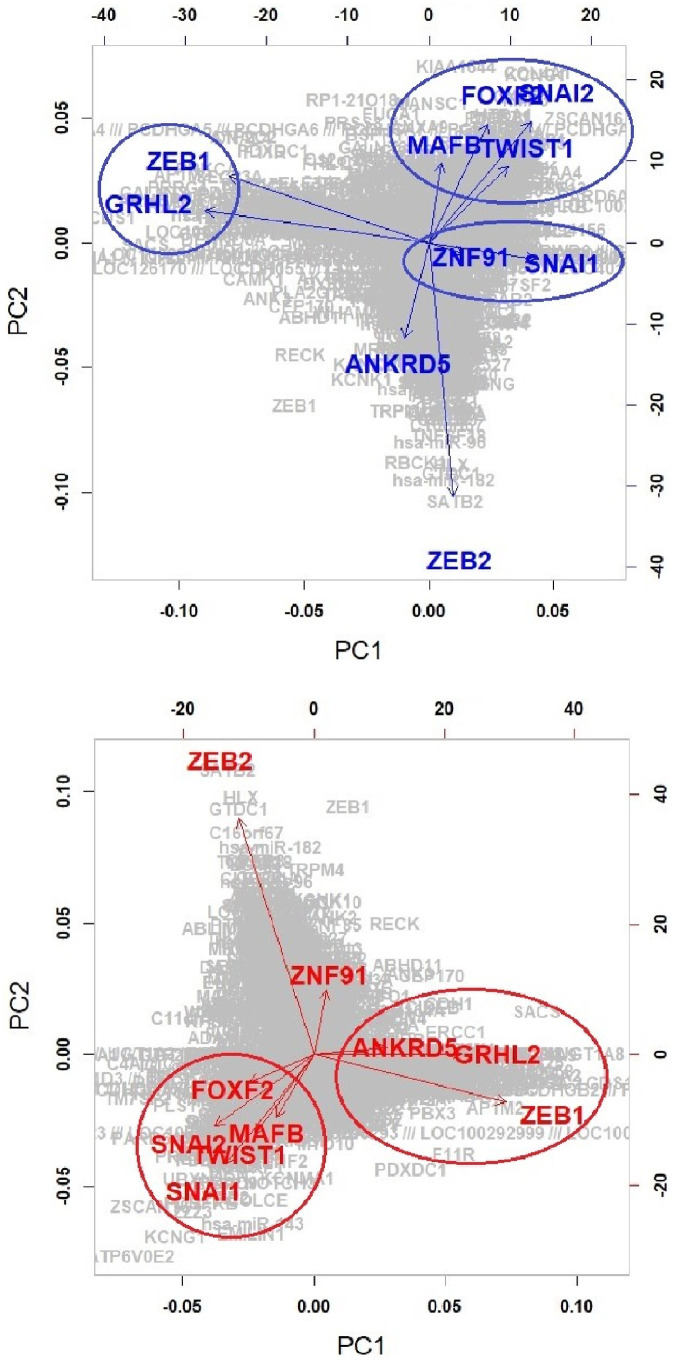
Projection of the discovered genes onto the first two principal components of REC.

Fig 6 shows the projected 10 genes and 13,493 target genes on the first two PC spaces of the REC matrix. From the biplot, the identified genes can be grouped as follows,

**High.E&High.C1 region**
  - Group 1: ZEB1, ANKRD5, GRHL2
  - Group 2: FOXF2, MAFB, SNAL1, SNAL2
**Low.E&Low.C1 region**
  - Group 1: ZEB1, GRHL2
  - Group 2: FOXF2, MAFB, TWIST1
  - Group 3: ZNF91, SNAL1

We focus only on the newly discovered five genes because the reciprocal mechanisms between the five EMT-TFs were well known in previous studies. For both high and low regions of the first component, GRHL2 is grouped with well-known EMT marker ZEB1. Their reciprocal mechanisms in the EMT-related process are demonstrated as follows [25, 27, 28]: GRHL2 suppresses EMT and restores sensitivity to anoikis by repressing ZEB1 expression; Combination of TGF-*β* and Wnt activation represses GRHL2 expression by direct interaction of ZEB1 with the GRHL2 promoter, inducing EMT; Reciprocal feedback loop between GRHL2 and ZEB1 controls epithelial versus mesenchymal phenotypes and EMT-driven tumor progression; GRHL2 is the main gatekeeper of EMT in EOC via miR-200-ZEB1, and their axis forms the core of EMT signaling. The EMT-related interaction between FOXC2 and TWIST1 is also demonstrated as follows [21, 22]: FOXC2 transcriptionally represses the expression of two EMT-TFs TWIST1 and FOXC2; FOXC2 negatively targets TWIST1 in the EMT programming and metastasis progress of BLBC. Genes MAFB and ZNF91 are members of Gene-Ontology-Terms Class: GO:0006355—regulation of transcription, DNA-templated. However, the interaction between MAFB and ZNF91 in diseases has not been yet demonstrated.

**Component 2**:

**High.E&High.C2 vs High.E&Low.C2**

**Low.E&High.C2 vs Low.E&Low.C2**

For the second component, we consider regions of high and low values of the second component for high and low EMT modulators, respectively (i.e., “High.E&High.C2 (195 cell lines) vs High.E&Low.C2 (186 cell lines)” and “Low.E&High.C2 (186 cell lines) vs Low.E&Low.C2 (195 cell lines)”). In addition to the well known five EMT-TFs, GLI3, ANKRD5, PCBD1, FOXF1, OVOL2 and LSR, TP63, SOX13, IFI16, and TGFB1I1 are identified for low and high EMT regions, respectively.

**Fig 7:**
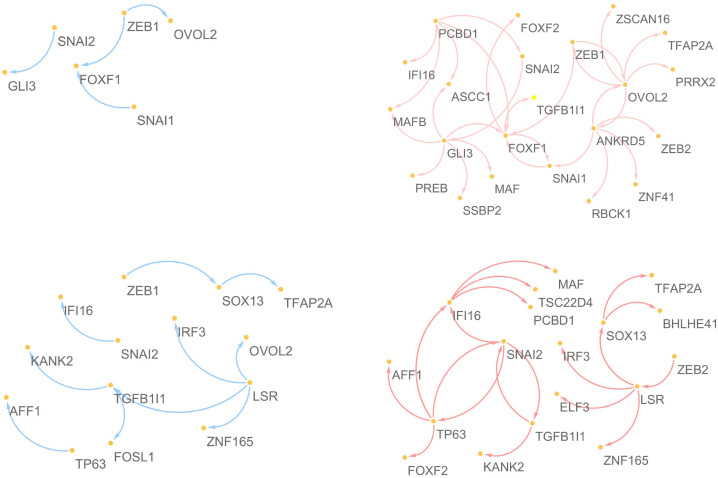
Target networks of the five regulators discovered from the analysis of the second component for high and low EMT regions.

**Fig 8:**
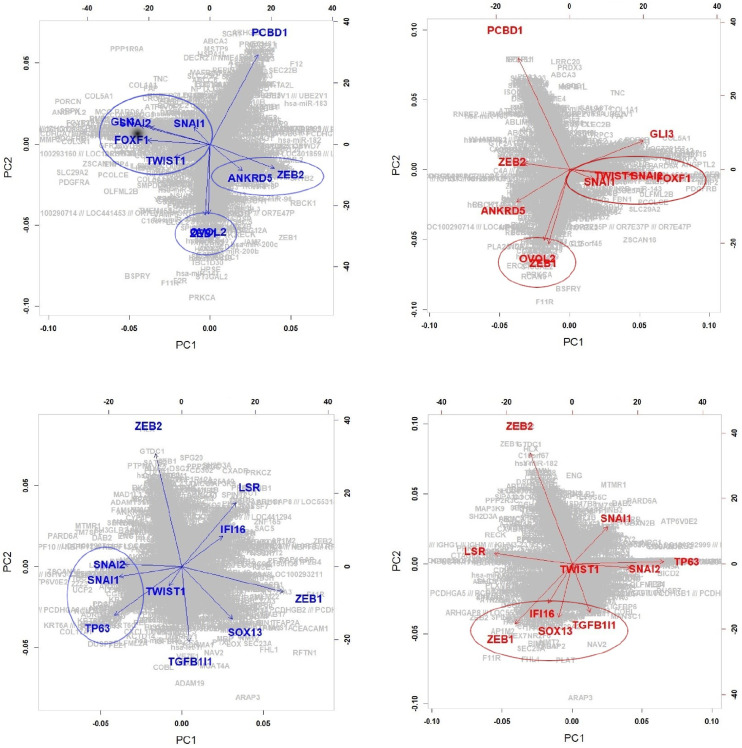
Projection of the discovered genes onto the first two principal components of REC: Component 2.

Fig 7 shows target networks of the newly identified five markers other than 5 EMT-TFs, where the edges appeared in each network of all cell-lines are only extracted. Similar to the component 1, a relatively sparse network can be seen in the low regions of component 2, as compared to the high regions. Especially in the low EMT region, it can be seen that the target network consists of only well known EMT markers, ZEB1, SNAI1, SNAI2, OVOL2, FOX1 and GLI3 in region for the low component 2, whereas the target network for the high component 2 involves many genes. Fig 8 shows the results of PCA for the REC matrices. We focus on the results of the low EMT region (i.e., Low.E&High.C2 versus Low.E&Low.C2) and group the genes as follows,

**ZEB1 and OVOL2**: OVOL2 is one of the well-known EMT markers, and the interaction in the EMT process of OVOL2 and ZEB1 has been demonstrated in many studies: OVOL-TFs control MET through a regulatory feedback loop with EMT-inducing TF, ZEB1 [41]; OVOL2 restricts EMT by directly inhibiting EMT-inducing factors including the ZEB1 system; A regulatory network containing OVOL2–ZEB1 mutual repression results in a four-state EMT, i.e., epithelial, intermediate, intermediate, and mesenchymal states [42]; OVOL2 suppresses ZEB1 expression by binding to the ZEB1 promoter [39].
**FOXF1 and SNAI1**: FOXF1 is also confirmed as an EMT-related marker, and reciprocal mechanisms of genes in this group have been demonstrated: the expression of FOXF1 inhibits cancer cell invasion and migration, whereas the inactivation of FOXF1 stimulates cell invasion and migration (Wei et al., 2014); higher level of FOXF1 is positively associated with enrichment of EMT gene signatures [19]; Overexpression of FOXF1 induces EMT by transcriptionally activating SNAI1 in colorectal cancer metastasis [19].
**GLI3 and SNAI2**: GLI3 is one of the zinc finger protein and well-known marker of Sonic Hedgehog, and interaction between GLI3 and SNAI2 has been demonstrated: shRNA-GLI3-transfected cells were associated with the decreased expression of stem cell- and EMT-related genes (CD44, BMI1, POU5F1, and SNAI2) [24].

**Component 3**:

**High.E&High.C3 vs High.E&Low.C3**

For the third component, we consider High.E&High.C3 and High.E&Low.C3 regions consisting of 154 and 227 cells, respectively. TP63, IFI16, AFF1, IRF6, and KANK2, other than EMT-TFs, show significantly different edges between the High.E&High.C3 and High.E&Low.C3 regions, where TP63 and IFI16 are also identified as crucial genes in the analysis of the second component. IRF6 is one of the 50 EMT-related genes defining the EMT modulator. The EMT-related mechanism of IRF6 has been demonstrated in previous studies; especially, there are many studies on the association between well-known EMT markers with IRF6: ectopic expression of IRF6 increases the expression of SNAI2 and diminishes the expression of various epithelial markers (e.g., E-cadherin) in EMT; TGF*β*3 increases IRF6 expression, and IRF6 appears to regulate EMT during palatal fusion via SNAI2 [33]; IRF6 is downregulated during the EMT process of breast cancer and prostate cancer [34]. Although EMT-related mechanisms of KANK2 have not yet been revealed, it has been demonstrated that KANK2 concentrates around most mature focal adhesions and binds talin in migrating cells [51]. AFF1 is known as the mixed lineage leukemia fusion-associated gene and plays a role in osteogenic differentiation of human mesenchymal stem cells [52–54]. However, EMT-related mechanisms of AFF1 have not yet been demonstrated.

**Fig 9:**
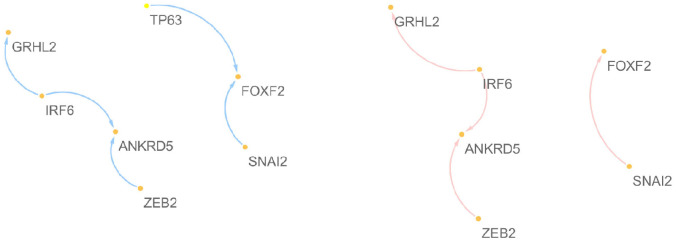
Target networks of the five regulators discovered from the analysis of the third component.

The target networks of the newly identified five genes are presented in Fig 9. There are no significant differences between the two regions, except for the association between TP63 and FOXF2. It can be considered that the target networks related to the third component may be dominated by the mechanism of the high EMT region, and only regulating FOXF2 by TP63 can be considered as a specific characteristic related to component 3.

**Fig 10:**
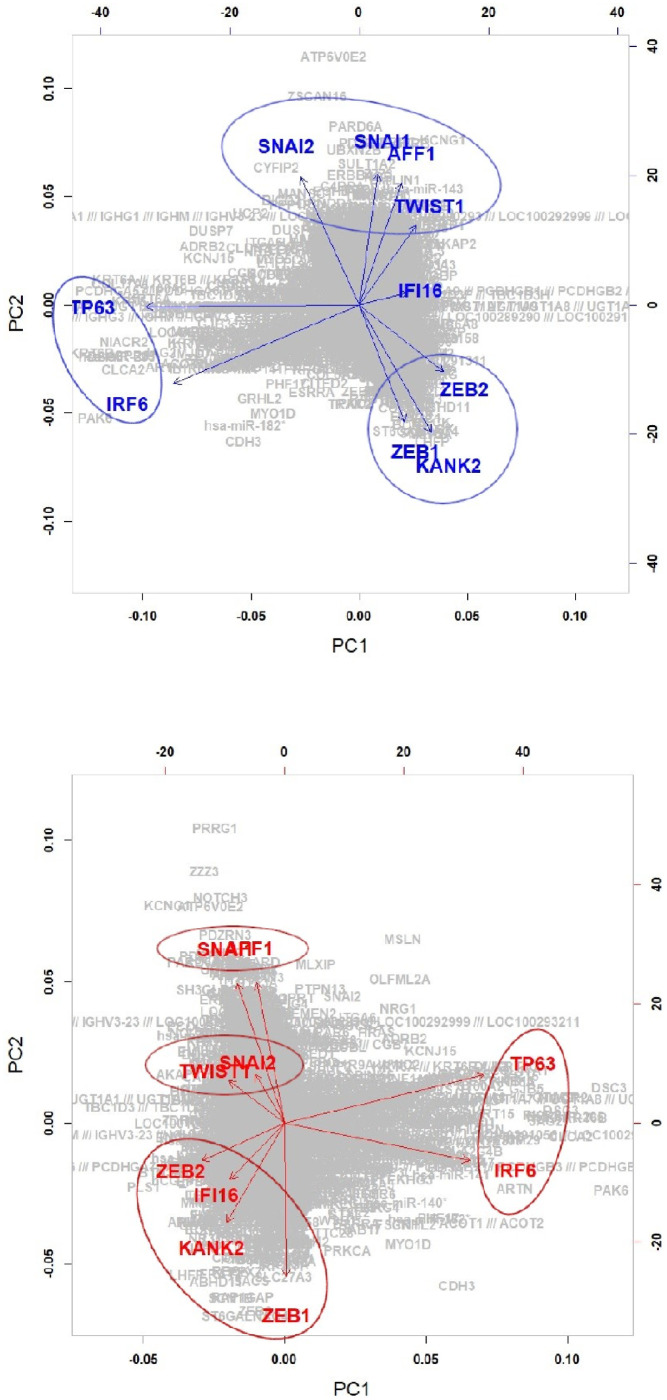
Projection of the discovered genes onto the first two principal components of REC: Component 3.

Fig 10 shows the projected 10 regulators and target genes on the first two PC spaces of REC. We group the genes as follows,

**Group 1**: **IRF6 and TP63**
  IRF6 regulated by TP63 plays a tumor suppressor role in squamous cell carcinomas through a Notch-dependent mechanism, which plays critical roles in EMT pathway [34].
**Group 2**: **AFF1 and SNAI1, SNAI2, TWIST1**
**Group 3**: **KANK2 and ZEB1, ZEB2**

The reciprocal mechanisms of genes in groups 2 and 3 have not yet been demonstrated.

### Functional enrichment analysis based on the bioinformatics tool DAVID

**Fig 11:**
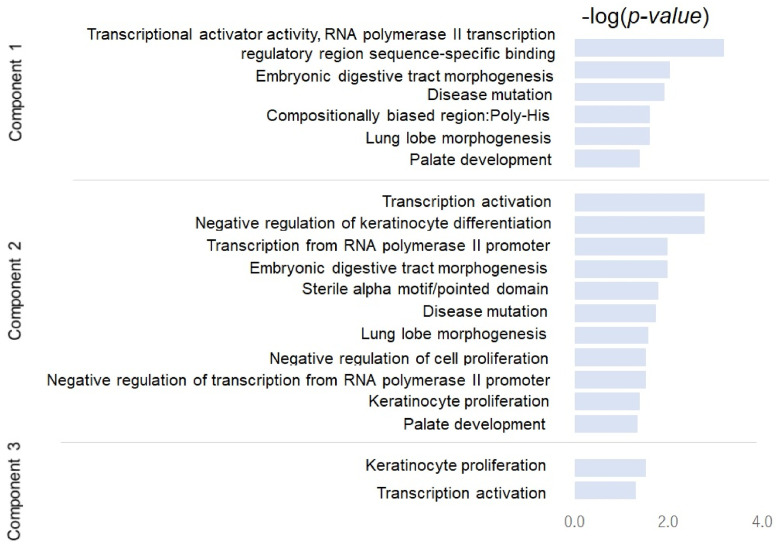
Gene enrichment analysis of the identified crucial genes from the REC matrix.

To identify biological processes involved in the extracted components, we performed gene enrichment analysis using the bioinformatics tool Database for Annotation, Visualization and Integrated Discovery (DAVID) [55]. We used all regulatory genes as a background and performed functional enrichment analysis for 17 genes (1% of the regulatory genes) other than five EMT-TFs showing different regulatory systems in the high and low regions of each component. Fig 11 shows the functional annotation chart (*p* < .05) and the corresponding p-value (i.e., −*log*(*p*.*value*)). For the first component, six clusters are found, where the most significant cluster corresponding to the lowest p-value (i.e., the highest score for enrichment) is “*transcriptional activator activity, RNA polymerase II transcription regulatory region sequence-specific binding*” grouping genes OVOL2, MAFB, FOXF1, FOXF2, GRHL2, and ALX1. The “*RNA polymerase II transcription regulatory region sequence-specific binding*” is a GO annotation of ZEB1. “*Embryonic digestive tract morphogenesis*”, “*disease mutation*”, “*lung lobe morphogenesis*”, and “*palate development*” are common factors enriched in the first and second components. Except for “*disease mutation*”, the three clusters are the GO functional terms (http://www.informatics.jax.org/),

- Embryonic digestive tract morphogenesis (GO:0048566): the anatomical structures of the digestive tract are generated and organized during embryonic development. The digestive tract is the anatomical structure through which food passes and is processed.
- Lung lobe morphogenesis (GO:0060463): The process in which the anatomical structures of a lung lobe are generated and organized. A lung lobe is a projection that extends from the lung.
- Palate development (GO:0060021: Roof of mouth development): The biological process whose specific outcome is the progression of the roof of the mouth from an initial condition to its mature state. This process begins with the formation of the structure and ends with the mature structure. The roof of the mouth is the partition that separates the nasal and oral cavities.

We focus on *sterile alpha motif/pointed domain* (grouping FLI1, ELF3, and TP63), which is involved in interactions with proteins, DNA and RNA. In a previous study, it was demonstrated that sterile alpha motif-pointed domain containing ETS TF (SPDEF) negatively regulates CCL2 and the EMT markers in prostate cancer cells, and the interaction between SPDEF and CDH1 (E-cadherin) related to the EMT process was also demonstrated: decreased SPDEF levels significantly induce CCL2 and CDH2 (N-cadherin), and decrease CDH1 (E-cadherin) mRNA and protein expression, confirming the association between SPDEF inhibition and EMT in cells. [56]. Although only ELF3 was demonstrated as a crucial regulator of E-cadherin in Shimamura et al., [1], interactions FLI1 and TP63 with E-cadherin are also verified as follows: Cav1-Snail-E-cadherin pathway plays a central role in the expression of the oncogenic transformation functions of fusion gene EWS/FLI1 [57]; reactivation of △*Np*63*a* is linked to the maintenance of epithelial markers and suggests that E-cadherin has a dual role in lung squamous cell carcinoma [58].

For the second and third components, *“transcription activation” (grouping TP63, TGFB1I1, and GRHL2)* and *“keratinocyte proliferation” (grouping IRF6 and TP63)* are the most enriched terms, respectively. *keratinocyte* is frequently reported to be related to the second and third components in Fig 11. *keratinocyte* plays a role in cell–cell adhesion [59, 60]. We found that gene TP63, which is known as a keratinocyte TF, plays a key role in the EMT process through interaction with the well-known EMT markers, i.e., TGF-*β*, GRHL2, and miR-200n family: Ectopic △*Np*63*a* expression in normal human epidermal keratinocytes yields the EMT phenotype in a TGF-*β*-dependent manner; Knockdown of all isoforms of p63 leads to the EMT phenotype through loss of GRHL2 and miR-200 family genes [61].

Furthermore, it has been found that IRF6 (target of TP63) is induced by the NOTCH signaling pathway, which plays vital roles in the development and progression of cancers through regulating ZEB1 expression and EMT pathway, in breast cancer and keratinocytes [34]. Interestingly, the cluster consisting of IRF6 and TP63 related to keratinocyte proliferation was also identified by PCA of REC for the third component (see Fig 8). In addition, it was shown that the interaction of TP63 and FOXF2 is uniquely different in the target networks for the high and low regions of the third component (see Fig 7). In short, the third component and a part of the second component of the EMT-related networks can be described as a keratinocyte-related factor and the interaction of TP63 and IRF6, which may play a vital role in EMT in keratinocytes.

## Discussion

We introduced a novel methodology for a comprehensive analysis of large-scale personalized network tensors. In this study, we considered a gene regulatory network as a tensor for a data point, and decompose the multilayer networks represented as tensors by using an AI approach, TRIP. Unlike existing studies for sample-specific gene network construction, our strategy analyzes whole multilayer networks based on tensor decomposition, thus we can perform wide exploration of the large-scale gene regulatory networks for all patients. To illustrate our method, we applied it to personalized networks constructed for 762 cell lines having varying conditions of the EMT process. We identified novel candidate markers and verified biological mechanisms of a majority of the identified markers based on the literature. Although most of the identified markers were found in previous studies, some of the revealed genes could not be verified. Further work is required towards experimental validation of the newly revealed markers. In this study, our strategy was illustrated based only on EMT-related gene regulatory networks. As one of our future works, we consider the comprehensive analysis of dynamic systems of personalized gene networks in accordance with various clinical characteristics (e.g., drug sensitivity of cell lines).

Global exploration of multilayer networks along with clinical characteristics of a patient provides crucial information for evidence-based personalized medicine. We expect that the proposed strategy based on an explainable AI approach will provide a novel insight into network biology.
