# Correction: Global gene network exploration based on explainable artificial intelligence approach

**DOI:** 10.1371/journal.pone.0246380

**Published:** 2021-01-27

**Authors:** Heewon Park, Koji Maruhashi, Rui Yamaguchi, Seiya Imoto, Satoru Miyano

The following information is missing from the Funding statement: This work was supported by MEXT as Innovative Areas (JP15H05912), “Priority Issue on Post-K computer” (Integrated Computational Life Science to Support Personalized and Preventive Medicine, Project ID: hp180198, hp190158), and “Program for Promoting Researches on the Supercomputer Fugaku” (Unravelling origin of cancer and diversity by large-scale data analysis and artificial intelligence technology, Project ID: hp200138). This work was also supported by JSPS KAKENHI (JP19K20402, JP18H03329).

An additional affiliation is missing for the third author. Rui Yamaguchi is also affiliated with Division of Cancer Informatics, Nagoya University Graduate School of Medicine, Aichi, Japan.
